# Should cognitive behavioral therapy for insomnia be considered for preventing and managing chronic pain?

**DOI:** 10.1093/sleep/zsae177

**Published:** 2024-08-02

**Authors:** David M Klyne, Simon S Smith, Michelle Hall

**Affiliations:** NHMRC Centre of Clinical Research Excellence in Spinal Pain, Injury and Health, School of Health and Rehabilitation Sciences, University of Queensland, Brisbane, QLD, Australia; Child Health Research Centre, Faculty of Medicine, University of Queensland, Brisbane, QLD, Australia; Sydney Musculoskeletal Health, The Kolling Institute, School of Health Sciences, University of Sydney, Sydney, NSW, Australia

There is no question that chronic pain is one of the world’s biggest health challenges and current efforts to reduce the enormous burden of this condition are failing [1, 2]. Of all the health problems that often coexist with chronic pain, poor sleep is one of the most frequent, reported by up to 90% of people with chronic pain [3–7]. Despite the extraordinarily high cooccurrence between the two, clinical approaches to prevent or manage chronic pain rarely, if ever, consider sleep. One intervention that targets sleep with potentially high capacity to influence pain is cognitive behavioral therapy for insomnia (CBT-I). CBT-I is the gold-standard first-line treatment for insomnia disorder [8–10], a condition that affects up to 30% of the general population (i.e. the most common sleep disorder) and an even greater proportion in chronic pain populations [11–15]. Relative to other sleep disorders, insomnia also shares many of the same physiological consequences and symptoms as general poor sleep [16, 17]. For this reason, CBT-I could be prescribed on a “do no harm” basis to improve overall sleep health regardless of a formal sleep disorder diagnosis [18]. One must then ask, why is CBT-I not considered a clinical treatment option for individuals with chronic pain who sleep poorly [19]? The answer to this question depends on two further questions: (1) how strong is the evidence that CBT-I mitigates pain and (2) how would CBT-I optimally “fit” into current treatment plans to enhance the prevention and management of chronic pain?

Addressing the first question is not straightforward as most clinical trials on CBT-I have not considered pain as an outcome measure. Of those that have, the impact of CBT-I on chronic pain has usually been interpreted from self-reported pain measures as part of a battery of secondary outcome health questionnaires assessed prior to, during and/or after a course of CBT-I (for a review of some of these trials, see [20]). Our own preliminary synthesis of CBT-I trial data (six trials [21–26]) from 531 participants with chronic musculoskeletal pain demonstrates that CBT-I reduces pain (standardized mean difference of 0.43 [95% CI: 0.19 to 0.67], Figure 1). Furthermore, a meta-analysis of the effectiveness of CBT-I in patients with comorbid insomnia and chronic non-cancer pain showed that a reduction in pain was ~60% more likely immediately and 12 months after a course of CBT-I [27]. It is important to recognize that CBT-I is comprised of several components (e.g. stimulus control, relaxation, cognitive restructuring, and sleep hygiene), and each of these alone is capable of improving sleep in nonclinical populations [18]. This suggests that the pain-relieving benefits of CBT-I, which incorporates all these components, might also extend to individuals with poor sleep health but who may not have a clinical diagnosis of insomnia. Collectively, these data support the hypothesis that improvements in sleep through CBT-I can meaningfully reduce chronic pain. However, this interpretation will require human trials that robustly test the clinical efficacy of CBT-I with “pain” as a primary outcome measure in populations with both chronic pain and insomnia disorder or “general” poor sleep health. Such trials would benefit from implementing full telehealth or hybrid telehealth/in-person (relative to traditional face-to-face CBT-I) digital delivery models to overcome accessibility and cost barriers that would typically limit clinical translation. Advances in telehealth technologies coupled with new evidence that telehealth-delivered CBT-I is as effective as in-person-delivered CBT-I at improving sleep [28] support this model of care.

**Figure 1.**
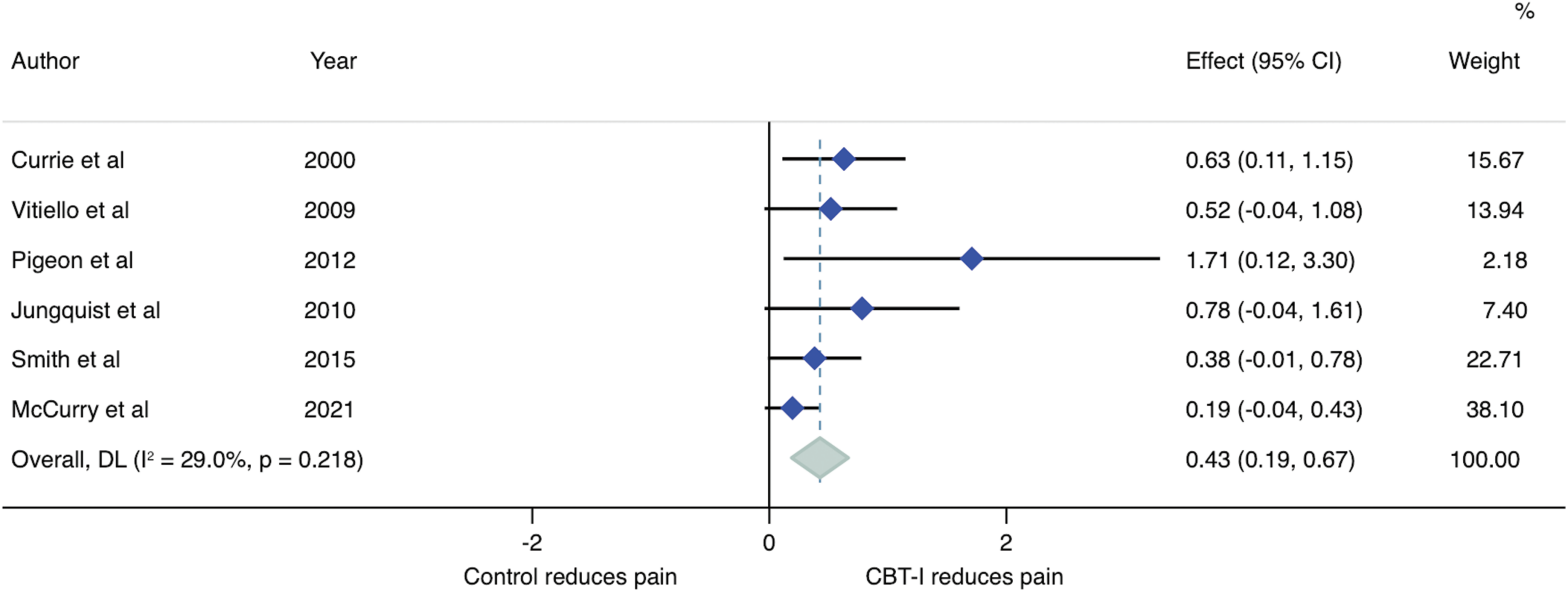
Standardized mean difference in pain immediately following cognitive behavioral therapy for insomnia (CBT-I) compared to usual care or no intervention in people with chronic musculoskeletal conditions.

The second question has received even less attention and will rely on future trials evaluating the analgesic effectiveness of CBT-I applied alone versus CBT-I combined with other interventions. Interestingly, CBT-I is thought to partly evoke its effects on pain via similar mechanisms to that of aerobic exercise, which is one of the most effective first-line treatments for reducing pain [29]. Both treatments can positively impact factors across domains of biology, psychology, sociology, and behavior such as mood, self-efficacy, socialization, fatigue, stress, diet, and sleep. Changes in each of these factors can evoke an array of physiological responses that could impact pain. With respect to neuroimmune circuitry, many of these responses are thought to converge into a common pathway: reduced inflammation [30–32] leading to changes in the peripheral and central nervous systems that lower pain [4, 32–35]. Insight into this common pathway was provided by our recent preclinical work in rats exposed to either *poor sleep*, *aerobic exercise* (wheel running), or *both* for 4 weeks after acute injury [34, 36]. As expected, poor sleep enhanced forepaw sensitivity and was accompanied by a systemic increase in brain-derived neurotrophic factor, which is triggered by inflammatory factors (e.g. tumor necrosis factor-α and interleukin-1β [37–41]) and contributes to pain sensitization via its capacity to regulate synaptic plasticity [42–45]—whereas exercise had the opposite effects. These findings corroborate our extensive work in humans [46–55]. Strikingly, however, concurrent exposure to both experimental conditions (poor sleep and aerobic exercise) evoked comparable effects to exercise alone—hinting that aerobic exercise counters the effects of *poor* sleep on pain via similar pathways, but oppositely. These data further suggest that, like exercise (and opposite to poor sleep), *improved* sleep alleviates pain, at least in part, via this same “neuroimmune” pathway, and that their combination could have an additive effect on pain reduction. The combination of aerobic exercise and improved sleep via CBT-I might also benefit pain through changes that are primarily evoked by one treatment but not the other. For example, we and others have shown that muscle strength, body weight, and fear of movement are important mediators of musculoskeletal pain relief in response to exercise [56, 57]. These mediators are unlikely to be directly affected by CBT-I. On the other hand, some psychosocial factors linked to pain such as interpersonal functioning may be more responsive to CBT-I than to exercise [58]. Although the case for combining CBT-I with aerobic exercise is developing, other routine interventions for pain may also synergize with CBT-I to promote greater pain relief. For example, like aerobic exercise, weight loss [59–62], dietary [63–66], short-term anti-inflammatory medication [67, 68], and various pharmacological and non-pharmacological psychological (e.g. targeting stress and depression) [69–73] interventions have known anti-inflammatory effects that can contribute to pain relief. In addition, like CBT-I, many of these same treatments enhance sleep quality via direct and/or indirect mechanisms (e.g. [74–77]), thereby potentially further amplifying the cycle of improved sleep, immune activity, and pain relief. Whether true and to what extent will have important implications for the potential future use of CBT-I to treat or even prevent chronic pain.

Moving forward, several clinical factors will need to be considered and tested in order to optimize CBT-I as a tool for preventing and/or managing chronic pain. These include the timing (e.g. after pain onset: acute vs. subacute vs. chronic phase) of application of CBT-I and the source (e.g. damage/injury to muscle, nerve, or bone) and/or type (i.e. nociceptive vs. nociplastic vs. neuropathic) of pain, all of which could impact the effects of CBT-I on pain. For instance, our ongoing work implies that central sensitization—a phenomenon of increased neuronal responsiveness in central pain pathways [78]—plays a critical role in the transition from acute to chronic pain, but that the rate and degree of central sensitization depends on the initial cause (e.g. non-nerve vs. nerve damage), which dictates the optimal timing for treatment initiation and type [30, 31, 34, 36, 42, 79]. Moreover, sleep might have a greater impact on pain that is predominately driven by peripheral tissue inflammation (e.g. as occurs with muscle, tendon, ligament, or bone trauma), where systemic inflammation can have a major impact [32], compared to pain that is predominately driven by nerve inflammation (as occurs with nerve injury), which is less dispersed and impacted by systemic changes. And, of course, various pain and/or non-pain factors might limit or completely negate the impact of CBT-I on sleep. Confirmation of these and other key clinical factors will require exploration of mechanisms under controlled sleep and injury/pain conditions, which would guide the likely optimal manner by which CBT-I could be applied in target pain populations in future clinical trials.

## Data Availability

Data from studies that are mentioned and owned by either Drs. Klyne, Smith or Hall will be made available following a written request to the corresponding author, along with a summary of the planned research.
